# Parent and Carer Skills Groups in Dialectical Behaviour Therapy for High-Risk Adolescents with Severe Emotion Dysregulation: A Mixed-Methods Evaluation of Participants’ Outcomes and Experiences

**DOI:** 10.3390/ijerph20146334

**Published:** 2023-07-10

**Authors:** Lindsay Smith, Katrina Hunt, Sam Parker, Jake Camp, Catherine Stewart, Andre Morris

**Affiliations:** 1 National and Specialist Child and Adolescent Mental Health Services (CAMHS), South London and Maudsley NHS Foundation Trust, Michael Rutter Centre, De Crespigny Park, London SE5 8AZ, UK; lindsay.smith@slam.nhs.uk (L.S.); katrina.hunt@slam.nhs.uk (K.H.); sam.parker@slam.nhs.uk (S.P.); jake.camp@slam.nhs.uk (J.C.); catherine.stewart@slam.nhs.uk (C.S.); 2 Department of Psychology, Institute of Psychiatry, Psychology & Neuroscience, King’s College London, London SE5 8AF, UK

**Keywords:** self-harm, suicidal behaviours, dialectical behaviour therapy, parents, carers, borderline personality disorder, emotional dysregulation, skills groups

## Abstract

Background: There is an established evidence-base for dialectical behaviour therapy for adolescents (DBT-A) in the treatment of young people with severe emotion dysregulation and related problems, including repeated self-harm and suicidal behaviours. However, few studies have reported on parental involvement in such treatments. This study aims to explore the outcomes and experiences of participants of a dedicated skills group for parents and carers embedded within an adapted DBT-A programme in the United Kingdom. Method: This study was conducted within a specialist outpatient Child and Adolescent Mental Health Services (CAMHS) DBT programme in the National Health Service (NHS) in London. Participants were parents and carers of adolescents engaged in the DBT-A programme. Participants attended a 6-month parent and carer skills group intervention and completed self-report measures relating to carer distress, communication and family functioning, at pre-intervention and post-intervention. Following the intervention, semi-structured interviews were also completed with a subgroup of participants to explore their experiences of the skills group and how they perceived its effectiveness. Quantitative and qualitative methods were used to analyse the data collected from participants. Results: Forty-one parents and carers completed the intervention. Participants reported a number of statistically significant changes from pre- to post-intervention: general levels of distress and problems in family communication decreased, while perceived openness of family communication and strengths and adaptability in family functioning increased. A thematic analysis of post-intervention interviews examining participant experiences identified six themes: (1) experiences prior to DBT; (2) safety in DBT; (3) experiences with other parents and carers; (4) new understandings; (5) changes in behaviours; and (6) future suggestions. Discussion: Parents and carers who attended a dedicated DBT skills groups, adapted for local needs, reported improvements in their wellbeing, as well as interactions with their adolescents and more general family functioning, by the end of the intervention. Further studies are needed which report on caregiver involvement in DBT.

## 1. Introduction

Dialectical behaviour therapy for adolescents (DBT-A) was developed for young people struggling with severe and pervasive emotional dysregulation, self-harm and suicidal behaviours [1,2] and is recommended in the UK as the treatment of choice for these difficulties [3]. DBT-A is an adaptation of DBT [4], which has been demonstrated to be effective for adults with a diagnosis of borderline personality disorder (BPD), which usually includes core problems with severe and pervasive emotion dysregulation, self-harm and suicidal behaviours [5,6]. DBT-A has been demonstrated to be more effective in reducing self-harm and suicidal ideation in emotionally dysregulated adolescents than “treatment as usual” (TAU) [7,8,9] and compared to a number of other interventions [10]. 

Families are known to play a critical role in supporting the development of functional emotional regulation strategies [11,12] and the burden of caring for a young person with this range of difficulties can be very high [13]. Early problematic patterns of interaction are a risk factor for emotional vulnerability and have been identified as significant determinants in the development of BPD. Maladaptive parenting, including parental hostility, overprotective and rejecting parenting styles and attachment disorganisation have been identified as strong predictors of the problems associated with a BPD diagnosis [14,15]. 

The theoretical underpinnings of DBT also highlight the importance of targeting the relationships between the individual and significant people in their environment. The biosocial theory [4] proposes that severe and pervasive emotion dysregulation (and related problems) develop and are maintained through the ongoing transactions between the individual’s biological vulnerability to high emotion sensitivity and the social environment’s invalidation of the individual’s emotional experiences. While the “social environment” may include many different sources of invalidation within the individual’s social network and wider community, the responses of those closest may be particularly significant in the maintenance of problems with emotion dysregulation and so teaching parents, carers and significant others skills to improve the quality of family interactions is potentially an important target for any DBT intervention [16]. 

Studies of the effectiveness of DBT interventions for family members has so far focused primarily on DBT-informed programmes (e.g., the Family Connections™ programme) with carers of adults with BPD. For example, Family Connections™ has been found to reduce burden and grief in family members of adults with BPD [17,18]. Studies of Family Connections™ adapted for caregivers of adolescents are currently more limited, though Boritz et al. [19] demonstrated similarly favourable responses in their evaluation of this programme with caregivers and family members of youths with mental health difficulties (though not specifically adolescents with emotion dysregulation, self-harm and suicidal behaviours). However, despite the positive adolescent outcomes for DBT-A and the significance of family interactions in its theory of change, research evaluating the experience and outcomes of parents and carers in DBT programmes for adolescents remains very limited.

In standard DBT-A, the primary mode through which parents and carers are involved in treatment is the multi-family skills group (alongside the other core treatment modes, which are explained in more detail below) [2]. The multi-family group format in DBT-A involves adolescents and parents/carers attending the same weekly skills class together, to learn and practice DBT skills with other families, facilitated by DBT therapists. Multi-family skills groups usually last around 2 h and include a mix of teaching, discussion and experiential exercises to promote skills acquisition for all participants. 

In the first study of parental outcomes in DBT-A, Woodberry and Popenoe [20] reported reductions in mean depression ratings for parents, however, for the majority their scores were not in the clinical range at the start of the intervention, raising the question of whether “depression” was a meaningful measure of change for this population. People who care for those with BPD/severe and pervasive emotion dysregulation show higher levels of psychological and somatic distress than the general population [21]. Given the unpredictability and potential risks faced in caring for someone with self-harm and suicidal behaviours [22], as well as the worry and sense of perpetual crisis [23], measures of anxiety and stress may more meaningfully capture the experiences and targets most relevant to parents in DBT-A programmes. Such feelings can also lead to significant problems in family communication and parent–child relationships [24,25]. Therefore, measures of family communication and quality of relationships may also be relevant to evaluations of DBT-A with parents and carers.

Flynn et al. [26] examined a potentially more meaningful range of outcomes of parents and carers attending a DBT-A multi-family group, as well as explored their experiences of the intervention qualitatively. Significant decreases were reported for burden, grief and parental stress post-intervention, though anxiety was not measured. Participants also reported that the skills component of the multi-family group had been helpful in addressing their own needs and the needs of their child. However, DBT-A aims to improve the quality of interactions between parents/carers and children and this study lacked a meaningful measure of changes in family relationships. Furthermore, in the qualitative branch of Flynn et al.’s [26] study, participants reported that their experience would be improved by the inclusion of a separate forum where they could openly reflect on their experiences without concerns about the impact of their disclosures on their child. It is also important to note that while the treatment developers of DBT-A designed a multi-family group format for the delivery of the skills training component, they allow for other skills training formats [2]. DBT programmes in the community sometimes need to make pragmatic adaptations—for example, in their community-based RCT, Santamarina-Perez et al. [9] opted for separate parent/carer and children’s skills groups due to resource constraints. No study to date has evaluated the outcomes and experiences of parents and carers in DBT who attend a skills group separate to that of their children.

Further evidence pertaining to the impact and format of carer involvement in DBT treatment for adolescents is clinically important for mental health services attempting to provide effective, comprehensive interventions whilst under ongoing financial and capacity pressures. The current study aimed to evaluate a parent and carer DBT skills training group, embedded within a DBT-A programme, as it was routinely delivered in a Tier 4 CAMHS outpatient DBT service for adolescents in a London-based National Health Service (NHS) organisation. There were two objectives: first, to assess whether parent and carer wellbeing and family communication improve from the start to the end of the intervention; second, to explore parent and carer experiences of the group, including reflections on potential mechanisms of change and the ways in which this particular group format and structure could be improved.

## 2. Materials and Methods

### 2.1. Setting 

The National and Specialist CAMHS DBT service at the Maudsley Hospital in London (UK) was established in 2009 to provide evidence-based interventions for young people with severe and pervasive emotion dysregulation, self-harm and suicidal behaviours and associated problems (see MASKED FOR REVIEW for further details). 

Inclusion criteria for adolescents to enter our DBT-A programme included: (1) aged between 13 years and 17 years and 4 months (at the point of referral), (2) one or more incidents of self-injury in the previous 6 months and (3) symptoms in at least a further four domains of the BPD subscale of the Structured Clinical Interview for DSM-IV [27]. While adolescents needed only one incident of self-injury in the past six months to meet the related inclusion criterion, in practice most had high levels of self-injury and suicidal behaviours, as this was a tier 4 setting (equivalent to an inpatient service). Exclusion criteria for the service included any of the following: a primary diagnosis of psychosis; substance dependency; any psychiatric disorder that needed more urgent assessment/treatment; the adolescent had previously opted out of the programme in the past three months.

Referrals are received from Tier 3 Community CAMHS and Tier 4 Adolescent Inpatient Units/Specialist Services, locally and nationally. The DBT programme delivers up to 12-month interventions for adolescents and their parents/carers, including the following treatment modes: weekly individual therapy sessions for adolescents, weekly skills training groups for adolescents and parents/carers (for the first 6 months of treatment), telephone coaching for adolescents and parents/carers (to promote skills generalisation) and a weekly team consultation meeting for the therapists (to provide support, problem-solving and to help maintain adherence to the treatment manual). Notably, the parent/carer and adolescent skills groups are run separately for resource reasons, which is different to the recommended multi-family group format in DBT-A.

### 2.2. Intervention

The parent/carer skills group attended in-person by participants in this study ran concurrently to the young people’s skills groups for the first 6 months of treatment (in three rounds of 6–7 sessions). The group was run as an open group with new participants accepted at the beginning of each round. The group (summarised in Table 1) provided psychoeducation, training and rehearsal of DBT skills and some space to share personal experiences of managing problem situations with young people at home (both for peer connection and for opportunities to try skills-based solutions with the support of peers and group facilitators). The structure of the group was adapted from available manuals for DBT [4] and DBT-A [2] and introduced parents and carers to the skills that adolescents learnt in their skills training groups: distress tolerance, mindfulness, emotion regulation and interpersonal effectiveness. Additionally, the group included sessions to help participants orient to the biosocial theory of severe and pervasive emotion dysregulation, and to learn further key skills from Miller et al.’s [2] manual, such as validation, behaviour change principles, and others based on dialectics from the “Walking the Middle path” module.

### 2.3. Study Design

A mixed-methods approach was adopted, using quantitative and qualitative methodologies. To assess the changes in reported symptoms of depression, anxiety and general distress in parents and carers, as well as the perceived quality of family communication and relationships by the end of the intervention, self-report questionnaire data were collected at the start and upon completion of the 6-month group programme (pre–post design). Additionally, a subsample of parents and carers who completed the group were invited to take part in semi-structured interviews exploring parent and carer experiences of the group.

### 2.4. Participants

Inclusion in the study was based on being a parent or carer of an adolescent engaged in the DBT-A programme and attending the parent and carer skills group component between 2016 and 2018. Due to the inclusion criteria for entry into the programme, this meant that all participants were in a primary caregiving role for an adolescent with self-harm/suicidal behaviours, emotion dysregulation and related problems. Parents and carers who were not available to start attending the parent and carer skills group were excluded from the study, as were non-completers on the basis of either their own or their child’s non-attendance or their child exiting the programme earlier than expected.

On this basis, 81 parents/carers initially met criteria for inclusion in the study. Twenty-nine participants were classed as non-completers and were therefore excluded. Of the 52 participants who completed the programme, complete datasets were available for 41 participants at the end of the intervention, resulting in a 21.2% attrition rate at post-intervention.

For the qualitative component of the study, parents/carers who had completed the 6-month group programme were invited to participate. Of these, 8 parents and carers consented to participate in a semi-structured interview, conducted in-person. Please see Table 2 for participant socio-demographics for the respective substudies. (See Table 2 for a summary of the socio-demographic characteristics of participants in both the quantitative and qualitative branches of the study).

### 2.5. Therapists

All parent and carer skills group facilitators were therapists with a core mental health professional training (e.g., clinical psychologist, family therapist, mental health nurse) who had also completed an intensive, foundational training in DBT with a licensed provider. Therapists accessed support and supervision via the weekly DBT team consultation meeting and their own regular professional supervision.

### 2.6. Procedures

In the quantitative branch of the study, data were collected from participants at the first parent/carer skills group session, representing baseline (T1), and at the final session 6 months later, representing post-intervention (T2). A programme therapist was available to all participants in case they experienced any emotional distress in completing the measures.

In the qualitative branch semi-structured interviews (of 45–60 min duration) were conducted with participants by an external clinical nurse specialist who had knowledge of DBT but no direct clinical involvement with the intervention. Participants were interviewed individually using a semi-structured interview schedule that was designed to elicit participant reflections about their experiences of the group, to explore whether participants report aspects of the group to have led to any changes and to explore any aspects of the group that participants felt might present opportunities for improvement and development.

### 2.7. Measures

The Hospital Anxiety and Depression Scale (HADS) [28] is a 14-item self-report measure of psychological distress. Subscale scores range from 0–21, with higher scores representing greater distress. The scale was designed for use in the general population and has been used widely in parents/carers of children with long-term conditions [29]. 

The Parent Adolescent Communication Scale (PACS) [30] is a 20-item self-report measure of the perceived quality of communication between an adolescent and their parent, in the present study completed from the parent/carer perspective. The dimension of “openness” includes positive aspects of communication and satisfaction of the parent/carer on the quality of communication. The dimension of “communication problems” refers to the parent/carer’s perception of aggressiveness and avoidance in communication. Higher scores indicate better parent–adolescent communication for both dimensions. The PACS has been shown to be a reliable and valid measure, with utility for evaluating family-focused interventions [31,32].

The Systemic Clinical Outcome and Routine Evaluation (SCORE-15) [33] is a self-report measure designed to indicate crucial aspects of family life that are relevant to the need for therapy and for therapeutic change. It has fifteen Likert scale items and six separate indicators, three of them qualitative, plus demographic information. Three subscales assess: (1) disrupted communication, (2) strengths and adaptabilities and (3) being overwhelmed by difficulties in family interactions. Lower mean scores (range 1–5) indicate higher perceived family functioning on all subscales. The SCORE-15 has shown good reliability and validity as a measure of family adjustment [34].

### 2.8. Data Analysis

To assess for change in scores on quantitative measures of parent/carer wellbeing and family communication taken at the start and end of the group, mean differences were compared using the paired samples *t*-test and relevant assumptions were checked. Power analyses indicated that a total sample size of *N* = 34 would have 80% power to detect medium effect size differences [35] for the paired samples *t*-test with a two-sided significance level of 0.05. Data analysis was completed using SPSS 22.0 [36].

Thematic analysis (TA) [37] was selected as the most appropriate method for a theoretically flexible qualitative analysis of parent/carer interviews. This allowed for patterns within the data to be identified. The analysis was “data driven” in that themes were directly formed from the original data, using a semantic approach, as opposed to examined with reference to categories identified a priori [37]. The survey data were manually coded and analysed by the third and final authors in accordance with the six-phase procedure of TA [37]: “(1) reading and familiarisation; taking note of items of potential interest; (2) coding across the entire dataset; (3) searching for themes; (4) reviewing themes; (5) defining and naming the themes; (6) writing up a report to finalise the analysis” [pp. 202–203].

Throughout the data collection and analysis phases, the third author used a reflexive process which included writing a journal, reflecting on their personal biases and accessing consultation when required. 

### 2.9. Ethics

Ethical approval to conduct the study was granted by the local Clinical Audit and Service Evaluation Committee within South London and the Maudsley NHS Foundation Trust (SLaM). Group participants were made aware that the measures would be used for the purposes of evaluating the outcomes of the group, that there was no obligation to complete the measures and that non-participation would not affect the treatment provided. Those individuals who chose to take part in the qualitative interviews provided additional written consent for their participation in the evaluation.

## 3. Results

### 3.1. Quantitative Findings

A total of 41 participants completed the HADS, SCORE-15 and PAC measures prior to starting the parent and carer skills group (time point 1) and at the end of their final session (time point 2). 

Mean HADS scores for both depression and anxiety subscales reduced significantly from time point 1 to 2 with medium effect sizes [35] (see Table 3). Mean depression scores at both time points were within the “non-clinical” range. Mean anxiety scores at both time points fell within the “mild” range. The PACS mean total scores and subscale scores increased significantly from time point 1 to 2 with small to medium effect sizes. A significant increase on the SCORE-15 “strengths and adaptability” subscale was found with a small effect size. No significant changes were found on the “overwhelmed by difficulties” or “disrupted communication” subscales of the SCORE-15.

### 3.2. Qualitative Findings

**Figure 1:**
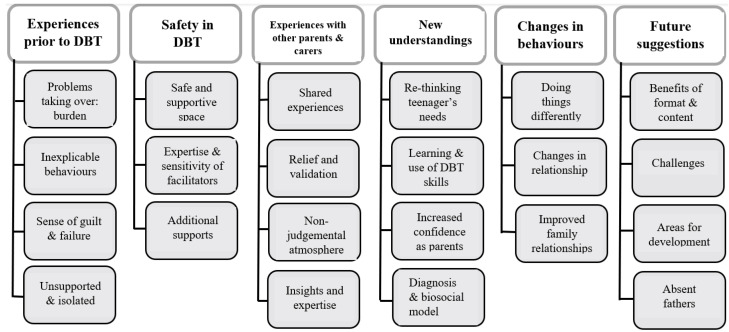
Themes and subthemes from the semi-structured interviews.

Analysis of the semi-structured interviews generated six themes and associated subthemes relating to parent/carer experiences in the group (see Figure 1).

Experiences prior to DBT

Each of the parent/carer participants brought reflections on family life prior to their child’s referral onto the DBT programme, even though there were no direct questions that explored experiences prior to DBT. This information seemed helpful to contextualise family life pre- and post-intervention.

Participants described a worsening situation managing the challenges of a child with this range of problems, which took on an increasingly central and destructive place in family life: “we just felt like we’d been shattered apart [...] my son became very angry, my husband was just completely stressed and withdrawn, and he wasn’t able to work for about six months” [P2].

Participants described feeling overwhelmed, frightened and confused as they tried to control hazards and manage behaviour they saw as extreme, volatile and inexplicable: “Chaos! A lot of anger and tensions; and treading on eggshells, worried if you say the wrong thing, or done the wrong thing. Lots of sleepless nights about whether you’re going to end up in A&E [...] there was a sense that it was out of your control” [P8].

Many described becomingly increasingly isolated and feeling unsupported by friends, family and local services: “our social circle…had diminished [...] so we had reached this sort of very insular state as a group. Our focus was purely on making sure [she] was safe” [P1].

Safety in DBT

All participants described feeling secure in the DBT treatment programme and within the groups and described finding a space to relax and reflect; this was partly because they felt their teenagers were “safe” in a concurrent group: “for the carers that might be the only [time] where they don’t have to worry about their child because their child is here […] and they can afford the luxury to actually think about it” [P5]; “…in here, you feel safe, because you feel people understand you and what you’re talking about” [P8].

Participants found it useful and containing to have professionals as facilitators, specifically professionals who they saw as experienced and able to answer questions but also who facilitated in a gentle and non-judgmental way: “the kindness of the conveners and their tact and the way that they responded and handled things was absolutely just excellent and really helpful and made it all feel just very sort of safe and just sort of a model of how to handle things” [P3].

Participants also highlighted that the commitment they and their child made to the DBT programme was an important part of them feeling safe: “The whole thing about DBT is that you kind of sign up to a contract so I felt safer” [P8].

Experience with other parents and carers

Participants had a universally and profoundly positive experience of being with other parents and carers. For some, this was about the sense of closeness that came from knowing they had been through similar challenges: “I just felt like I clicked with a lot of people as soon as I walked into the room, like, they get me, they get what I’m going through” [P5].

Some gained significant relief from feelings of guilt and isolation: “a great deal of comfort and understanding was gained from when somebody else would say: ‘my daughter does exactly the same thing’ so that is an immediate relief … that this feeling of isolation was beginning to be lifted” [P1].

Many participants said they found the experience of feeling listened to in the group validating, and for some this was also a cue for trying to do that more at home: “there was time in each group to talk about your feelings and everyone listened respectfully and thoughtfully and with helpful validations, and you can kind of get out of the habit of doing that at home when you’re fighting fires” [P2].

Participants also described benefiting from the insights and expertise of other parents: “I’d say the lessons that I’ve learnt have been as much from listening and talking to other parents and… [hearing about] their experience” [P4].

New understandings

In considering the impact of the group, all participants described an important change in the way they understood their teenager’s behaviours and needs. Several people described a shift to thinking more reflexively and from the teenager’s point of view: “I understand a bit more about what she’s perhaps going through [...] I never felt like that as a young person so I don’t quite get it but it gives me a way of trying to see things from her side” [P7].

Participants explained how this understanding led to changes in their own responses through the development of DBT skills: “Because of the mindfulness and all of these things that they teach us here—to take a step away, to really think, to take a mental break and all of that. It sounds really not like very much but it really helped us just to—just be lot more sensitive to how her days are” [P6]. Validation was frequently highlighted as a key skill that was helping them to make significant changes: “I found validation to be the concept and the skill that was most powerful and made sense of everything else—I could then see the point of everything else [...] when I get it right it makes a spectacular difference to my daughter” [P3].

Alongside skills acquisition, participants described increased confidence in themselves as parents, an ability to let go of past efforts to control behaviour and more willingness to trust their teenagers with responsibility: “I learnt that there isn’t a simple answer and you have choices [...] and that you do rely on your judgement and you can be flexible” [P3]; “going through the groups and things has made me realise that actually I’m not responsible for how he’s feeling. I can be there for him, I can support him, but he needs to know how to deal with how he’s feeling and the consequences of what he’s doing” [P5].

Participants highlighted the helpfulness of the biosocial model in gaining a better understanding of their child’s difficulties, though some also reported that while helpful this was an emotionally challenging part of the experience for them: “[the biosocial theory] was helpful to see, although painful […] recognising that you have a part in the environment and that influencing the super sensitivity that the child already has, I suppose it’s the regrets…[tearful]. The biosocial model was explained to us in a really sensitive way so… the sense of blame was pretty much from ourselves not from the facilitators at all […] So I found that session particularly tricky but that doesn’t mean that it was unhelpful” [P2].

Change in behaviours and relationships

Participants explained how conceptual shifts led to them experimenting with doing things differently and gave numerous examples of how they were responding more effectively to their children in everyday life: “It has made a difference in the way that I would react to what she’s done [...] I guess, your first reaction is to be angry, and… well, scared. Yeah, I guess, whereas now, we’re dealing with it rather than just getting that reaction” [P8].

Participants described the shared experience of going to the DBT skills group in parallel to their young person attending as opening up communication and connection: “having the group for the parents also means that the child thinks you know ‘oh that’s good because my mum’s going to be learning a bit about what I’m learning about at the same time’ and therefore it makes it easier for us to communicate I suppose” [P5]; “in the journey home in the car, and just reflecting on things we’d been learning about […] it kind of opened up a dialogue between all of us” [P2].

Participants spoke about positive changes in family relationships in terms of both reduced stress and increased connection: “the tensions were very high before. I think they’ve calmed down quite a lot” [P8]; “you’d see more communication now [...] we’re not as exhausted as we were then. My son’s not as angry as he was then […] that everything that was happening in the household was around his sister […] it’s a lot calmer, it’s a lot less stressed now [...] and it just slowly brought everybody back together” [P2].

Future suggestions for the group

Participants appreciated the structure and content of the group protocol and the opportunities to practice skills: “I thought it was very well structured […], it touched exactly on the things that parents worry about and that they struggle with every day” [P6]; “I thought the practical exercises were really good because it slows you down and makes you think about how ‘how would I actually try to use this?’” [P3].

In terms of challenges, participants discussed wanting a little more time in the weekly group sessions: “It might have been helpful to have a bit, maybe another half an hour because often you didn’t manage to finish or get through everything” [P7]. Some felt sad to leave the group and some felt anxious about how they would cope without it: “when I knew it was my last meeting, um, I felt quite ‘Oh God, I’m back on my own again!’ […] one mum actually sat there and cried on her last meeting because she said like: ‘You’ve just been so great, I feel so supported and now I feel like, you know, we’re not meeting anymore what am I gonna do?’” [P5].

Participants with other children reported that it would be helpful to have time devoted to helping them cope and balance their time as a parent between different children: “Help with helping siblings cope would be really, really helpful and I remember in one of the meetings one of the other parents said ‘oh couldn’t we have a group for children, for siblings?’” [P2].

A number of participants highlighted the absence of men/fathers in the group and linked this to issues of fathers sometimes being less involved and less present and the need to acknowledge that such “emotion-focused” spaces may be more difficult for men to access: “[my partner] didn’t come to any of the groups. Yeah. I mean it was more difficult for him because of work erm […] He never expressed really an interest to be honest in coming along […] I’ve always done all the things with the kids, it’s just an unspoken thing” [P7]; “…I felt that perhaps the father’s perspective in this field of varying degrees of emotional fluency or literacy was not sufficiently acknowledged” [P4].

## 4. Discussion

There remain very few studies that have reported parent/carer outcomes and the impact of parenting interventions as part of DBT for adolescents. This is despite there being a compelling case for involving family members in treatments that benefit both their own and their loved ones’ wellbeing [13,38]. The current study examined the preliminary outcomes and experiences of participants attending a structured parent and carer skills group within an adolescent DBT programme. Pre- to post-intervention scores on assessment measures revealed statistically significant reductions in parent/carer ratings of depression and anxiety and significant improvements in parent–child communication and family strengths. These results suggest that a skills group within a DBT-A programme that is specifically for parents and carers may be associated with improved outcomes for those caring for adolescents with emotion dysregulation, self-harm and related problems. However, future studies with control groups are needed to make conclusions regarding effectiveness.

Previous research has highlighted the critical role of primary carers in promoting the development of healthy emotion regulation in children [11,12] and the significant impacts experienced by parents and carers of adolescents with these problems [13]. Unhelpful parenting practice and attachment disorganisation have also been identified as strong predictors of a later diagnosis of BPD [14,15]. Therefore, it is extremely important to address the emotional and relational challenges faced by parents of children who self-harm [11,24,39] in the service of improving both their own and their children’s wellbeing. This is worth particular consideration given the transactional nature of the aetiology and maintenance of severe and pervasive emotion dysregulation combined with the potential benefits for family relationships when communication improves [16].

Flynn et al.’s [26] participants benefitted from attending a DBT-A multi-family group, in which adolescents and their parents/carers attend the weekly skills class together, though their participants suggested that a separate group for parents/carers could be more beneficial. Furthermore, resource issues sometimes mean a multi-family group format is not possible in a DBT-A programme [9]. The current study’s quantitative findings suggest that a separate parent/carer skills group within a DBT-A programme (running in tandem with a similar skills group for adolescents) may also be helpful for those attending, potentially helping to reduce parent/carer distress whilst simultaneously improving communication between parents/carers and their children and other aspects of family functioning.

The qualitative findings provide some further evidence for the benefits of skills groups for parents and carers in this context. The themes relating to “new understandings” and “changes in behaviour” indicate positive experiences of learning and implementing new skills, while themes of “safety” and “experience of other parents and carers” speak to the value of being able to connect as peers in a parent and carer skills group. Taken together, previous and current findings may suggest that both multi-family and parent/carer-only formats can be helpful for parents and carers in DBT-A programmes—the data providing some preliminary support for adapting the original DBT-A model in this way, when required.

It was of note in the current study, similar to Woodberry and Popenoe’s [20] findings, that participants’ depression scores were not in the clinical range before or after the intervention. This adds further weight to the question of whether depression is a meaningful measure of change for this population. Interestingly, the current study’s participants’ mean anxiety ratings *were* clinically significant at the start of the intervention, at the upper end of the “mild” range. While the significant decrease at post-intervention was not clinically significant, this finding indicates that parents and carers of children with high-risk behaviours may be more likely to struggle with anxiety-related problems themselves. This would make sense given the high levels of risk, uncertainty and potential threat that characterise the lives of carers of loved ones with a BPD diagnosis [21] or a similar range of problems. Certainly, participants in the current study described exactly these types of experiences and struggles, as reported in the “prior to DBT” theme. The current findings suggest that attendance at a parent and carer skills group may help to reduce prominent anxiety symptoms, in tandem with promoting improvements in family communication and functioning.

The results suggest that family communication and relationships between parents/carers and their children may significantly improve by the end of the intervention. Specifically, parents reported significantly fewer problems and breakdowns in communication and improvements in openness at the end of the skills group compared to the beginning. These outcomes are important to explore for the theoretical and conceptual reasons outlined above, but also given that previous studies have highlighted them as a neglected area in this field [23,25,40]. The positive outcomes demonstrated invite the question, which specific elements of the intervention may help with improving communication, and in what ways? Participant reports from the qualitative interviews give some insights into what may have been helpful. For example, within the theme of “new understanding” some discussed the importance of psychoeducation in providing alternative explanations for behaviours, which may support reductions in blame and criticism in parent–adolescent interactions.

Significant improvement was also reported at the end of the intervention in perceived family strengths and adaptability in problem solving and coping with challenges. This finding is consistent with the participants’ qualitative reports of the value of learning and using new skills, changing their own behaviour by doing things differently and developing a renewed sense of confidence in their parenting. By comparing DBT interventions with and without a skills training component, Linehan et al. [41] demonstrated that interventions that include DBT skills training are more effective for adults with a BPD diagnosis than DBT without skills training. This highlighted the potential importance of skills training for clients as a mechanism of change for adults with self-harm and suicidal behaviours. While outcomes for adolescents in the DBT-A programme were not examined in the present study, the findings offer some support for the value of parent/carer skills training within a DBT-A programme. This also raises the interesting question of the degree to which parent/carer skills development acts as a mechanism of change for adolescents in DBT-A, which may be a useful focus for future studies.

Some of the identified parent/carer improvements in this study may have also been influenced by the specific skills that participants acquired during the intervention. In their qualitative reports, participants identified a positive impact of skills learning, in particular mindfulness and validation. They also reported a positive impact of being able to better self-manage by using DBT skills for themselves. The importance of learning skills for carers has been identified in previous studies [42], and these findings also resonate with prominent hypotheses in the field of what maintains problems with severe and pervasive emotion dysregulation. Evidence suggests that difficulties with severe emotion dysregulation as experienced by many DBT clients develop in a complicated transaction in which high emotion vulnerability (e.g., sensitivity, reactivity, and a slow return to emotional equilibrium), growing pervasive emotion dysregulation and inaccurate expression (of emotions, wants, needs, etc.) elicit increasingly invalidating responses from the individual’s social and family environment and vice versa [43,44]. Dedicated parent and carer skills groups may help to equip participants to develop both the skills to manage their own emotions in highly distressing interactions with their children as well as the skills to validate, which in turn may help their children to regulate and communicate more effectively.

Finally, parents and carers in the current study highlighted some important suggestions for how their experience of such a group could be further enhanced. In the present study, less than a quarter of the skills group completers were fathers or male carers, and this imbalance and the need to address this were discussed in the qualitative interviews. The “absence of fathers” theme resonates with existing literature on the underrepresentation of fathers in parenting interventions [45] and family-focused therapies for children with mental health problems [46,47]. To date, there has been very little research conducted to understand these low rates of father/male carer participation and to facilitate the development of interventions to meet the needs of fathers/male carers specifically. However, Tully et al. [48] surveyed a large community sample of fathers who had been offered parenting interventions of various types. Participants reported a range of barriers to their engagement, including beliefs (e.g., “I don’t feel like my child’s behaviour is a problem” and “I don’t need help with my parenting”), work commitments and a lack of information about the effectiveness of the programmes. Further research is needed to better understand what might make it difficult for fathers and male carers to access the supports of DBT-A programmes.

### Strengths and Limitations

While the results of this study show promise, there are some notable limitations to be acknowledged. Firstly, as there was no control group, it is not possible to determine whether the changes observed were due to parents and carers participating in the skills group intervention or other factors (e.g., other elements of the DBT-A programme, the passage of time). Therefore, inferences regarding effectiveness cannot be made. Secondly, we must note that the data were collected within a busy routine service, with an opt-out rate of 36% and a data attrition rate at post-intervention for programme completers of 21.2%. Such limitations have prevented more rigorous statistical approaches like intention to treat analyses and mean the quantitative results, in particular, must be interpreted with caution. The potential for individuals with less severe problems and disrupted lives to have been more likely to participate presents a source of bias to the results and limits generalisability, but also therefore the data may underestimate the potential effect of the group. Thirdly, no measures or qualitative feedback were provided by adolescents relating to the involvement of their parents and carers in their treatment and any potential impact for themselves. Obtaining corroborative feedback from adolescents would be an important and helpful addition in future research. Follow-up measures were not taken and so it is not clear whether positive impacts of the intervention were sustained over a longer period. Lastly, we recognise that in TA, as in other qualitative methodologies, the researchers’ interpretation of the data is both an integral part of the paradigm and also potentially a limitation if that interpretation has moved too far from the data [49]. Whilst we employed reflexive processes and adhered to the TA phases, we acknowledge the potential for researcher bias in the interpretation of the data.

Further research is needed of parent/carer groups in DBT-A and parent/carer-focused aspects of the intervention more broadly. This would enable more in-depth investigation of the effectiveness of such interventions for parents/carers and young people in DBT-A programmes and potentially the opportunity to determine the specific mechanisms of change within DBT-A that are most influential in improving outcomes. It will also be important to further explore the experiences of parents and carers invited to DBT skills groups, particularly those who are underrepresented in such groups and those who may encounter barriers to accessing this potentially helpful element of the therapy.

## 5. Conclusions

Involving parents and carers in treatment for adolescents with severe and repeated self-harming and suicidal behaviours is known to be important in their recovery. Yet there is a paucity of research in this area, and previous studies had not explored outcomes related to family relationships and functioning. Additionally, some adolescent DBT programmes in the community face logistical challenges in delivering all aspects of the evidence-based DBT-A model, particularly the recommended multi-family skills group component. The current study provides evidence for the potential benefits of a dedicated and separate parent and carer skills group within an adapted DBT-A programme for reducing caregiver distress and improving family functioning. There remains a considerable need for further research on the effectiveness of methods of involving and supporting caregivers of adolescents who self-harm.

## Figures and Tables

**Table 1:** Parent and carer DBT skills group content.

Round	Week	Topic
1	1–2	Orientation/Biosocial Theory
	3–4	Understanding and Responding to Self-Harm and Suicidal Behaviour
	5–7	Distress Tolerance
2	8	Validation (1) (+mini-orientation)
	9–11	Interpersonal Effectiveness
	12–14	Emotion Regulation
3	15–17	Ways to Increase & Decrease Behaviours (+mini-orientation)
	18	Validation (2)
	19–21	Walking the Middle Path

**Table 2:** Parent/carer socio-demographic characteristics.

Characteristics	Quantitative Study %, (*N* = 41)	Qualitative Study %, (*N* = 8)
Gender of parent/carer:		
Female	76	75
Male	24	25
Gender of adolescent:		
Female	93	88
Kinship to adolescent:		
Mother	76	
Father	22	
Other family member	2	
Ethnicity:		
White British	68	88
White European	15	12
Black British/Black Caribbean	5	
Black African	5	
Asian	5	
Arabic	2	
Age range		
35–44	-	50 (4)
45–54	-	25 (2)
55–64	-	25 (2)

**Table 3:** Outcome measure means at pre- and post-intervention.

Measure	Group Mean (SD)		t Value	Cohen’s d
	Time 1 (*N* = 41)	Time 2 (*N* = 41)		
HADS Depression	7.44 (4.34)	5.39 (3.49)	3.80 ***	0.59
HADS Anxiety	10.71 (4.09)	8.49 (3.08)	4.30 ***	0.67
PACS Total	58.52 (12.51)	63.46 (13.19)	−3.55 **	−0.56
PACS Problems in Family Communication	27.21 (6.62)	29.15 (6.60)	−2.09 *	−0.33
PACS Open Family Communication	31.32 (8.23)	34.32 (8.09)	−4.10 ***	−0.64
SCORE-15 Strengths and Adaptability	2.45 (0.82)	2.22 (0.69)	2.19 *	0.34
SCORE-15 Overwhelmed by Difficulties	2.61 (0.93)	2.53 (0.78)	0.67	0.10
SCORE-15 Disrupted Communication	2.42 (0.78)	2.29 (0.56)	1.26	0.20

* *p* < 0.05, ** *p* < 0.01, *** *p* < 0.001; Cohen’s [36] effect size d: small ≥0.2; medium ≥0.5; large ≥0.8; HADS clinical cut-offs: 0–7 (Normal); 8–10 (Mild); 11–15 (Moderate); 16–21 (Severe).

## Data Availability

The data presented in this study are available on request from the corresponding author. The data are not publicly available due to the data being held within a clinical service and containing sensitive information.
